# Fingerprint analysis reveals sources of petroleum hydrocarbons in soils of different geographical oilfields of China and its ecological assessment

**DOI:** 10.1038/s41598-022-08906-6

**Published:** 2022-03-21

**Authors:** Qinglong Liu, Chunqing Xia, Lan Wang, Jingchun Tang

**Affiliations:** 1 College of Environmental Science and Engineering, Nankai University, 38 Tongyan Road, Jinnan District, Tianjin, 300350 China; 2 Tianjin Engineering Center of Environmental Diagnosis and Contamination Remediation, Tianjin, 300350 China; 3 Key Laboratory of Pollution Processes and Environmental Criteria (Ministry of Education), Tianjin, 300350 China

**Keywords:** Ecology, Environmental sciences

## Abstract

The distribution and characteristics of petroleum in three different geographic oilfields in China: Shengli Oilfield (SL), Nanyang Oilfield (NY), and Yanchang Oilfield (YC) were investigated. The average concentration of the total petroleum hydrocarbons (TPHs) conformed to be in the following law: SL Oilfield > NY Oilfield > YC Oilfield. Fingerprint analysis on the petroleum contamination level and source was conducted by the geochemical indices of *n*-alkanes and PAHs, such as low to high molecular weight (LMW/HMW) hydrocarbons, *n*-alkanes/pristine or phytane (C17/ Pr, C18/Ph), and ratio of anthracene/ (anthracene + phenanthrene) [Ant/(Ant + Phe)]. Soils adjacent to working well oils indicated new petroleum input with higher ratio of low to high molecular weight (LMW/HMW) hydrocarbons. The oil contamination occurred in the grassland soils might result of rainfall runoff. Petroleum source, petroleum combustion source, and biomass combustion were dominant PAHs origination of soils collected from oil exploitation area, petrochemical-related sites, farmland and grassland, respectively. The suggestive petroleum control strategies were proposed in each oilfield soils. Ecological potential risk of PAHs was assessed according to the toxic equivalent quantity (TEQ) of seven carcinogenic PAHs. The results showed that high, medium, and low ecological risk presented in petro-related area, grassland soils, and farmland soils, respectively. High ecological risk was persistent in abandoned oil well areas over abandoned time of 15 years, and basically stable after 5 years. This study can provide a critical insight to ecological risk management and source control of the petroleum contamination.

## Introduction

Petroleum hydrocarbons (PHs) are derived from animal and plant biomass by biogeological process through hundreds of millions of years, which have made a great contribution to the development of the economy^1^. However, human activities involving petroleum extraction, processing and transportation have caused serious petrochemical pollution to receiving environments^2^. Saturated hydrocarbons (SHs) and polycyclic aromatic hydrocarbons (PAHs) are the dominant components of PHs, especially PAHs as one of the persistent organic pollutants with two or more benzene rings have strong toxicity to the ecosystem and human health^3,4^. China has more than 700 oilfields, which distribute all over the country. Many studies researched the PHs content and components in different medium such as surface soil, sediment, oily sludge and groundwater in oilfield area. For example, the contamination status of PHs was researched in Yanchang Oilfield, a historic oilfield in northwest China^5^. The concentrations of PHs were measured in wastelands and sediments within the range of 1.67 × 10^3^–6.75 × 10^3^ mg/kg, and 1.19 × 10^3^–2.24 × 10^3^ mg/kg, respectively. The highest TPHs concentration was 1.83 × 10^5^ mg/kg in surface petroleum contaminated soil collected from Huabei Oilfield by previous study^6^. Most studies focused on the content distribution of total petroleum hydrocarbons (TPHs), however few studies have compared the component characteristics of petroleum hydrocarbons and derive source in different geographic oilfields. In view of the contamination caused by petroleum hydrocarbons, there was also a lack of regional pollution control management and potential ecological risk assessments based on pollution status in different oil fields.

The great geographic divergence on the petroleum hydrocarbons contamination was mainly due to human activities, physical and chemical properties of soils, local weathering and hydrogeological conditions, as well as the evolution process of petroleum hydrocarbons. Fingerprint analysis is an important technology to analyze geographic divergence of petroleum contamination based on the occurrence characters of petroleum components. The characters of petroleum components are the eminent indictors to reveal the contamination profile, pollution source, as well as evaluate the ecological potential risks. For example, low-molecular weight (LMW) PAHs such as 2–3-ring PAHs, originate primarily from the conversion of organic matter at low temperature and oil leakage; conversely, high-molecular weight (HMW) PAHs, such as 4-ring and higher PAHs, are derived primarily from burning fossil fuels at high temperatures^7,8^. Coastal soils taken from Persian Gulf were identified the sources and characteristics of PHs through fingerprint analysis based on the source-specific biomarkers of *n*-alkanes and PAHs isomers^9^. In Kareem reservoir, the information on source organic matters input, depositional environment, and maturation level were provided through analyzing the *n*-alkanes, isoprenoid alkanes such as pristine and phytane by using chromatography (GC) and gas chromatography–mass spectrometry (GC–MS)^10^. Pristine/phytane (Pr/Ph), Pr/nC17, and Ph/nC18 ratios indicated mixed sources of organic matters were dominantly with a less effect of biodegradation and mature stage of occurrence in their study.

Ecological risk derived from petroleum hydrocarbons especially PAHs caused widespread concern. PAHs have strong carcinogenic and teratogenic toxicity, and 16 of them are classified as priority control pollutants proposed by the US Environmental Protection Agency (EPA). Potential ecological risk index (RI)^11^, quality standard method (SQSs)^12^, toxic equivalent quantity (TEQ)^13^ were calculated to assess the ecological risk according to these priority control PAHs. Benlaribi et al. studied the risk assessment of polycyclic aromatic hydrocarbons in topsoils around a petrochemical industrial area in Algiers by TEQ index. They calculated TEQs showed the following trend: industrial-urban > urban > suburban > rural^14^. TEQ concentrations were calculated to assess the toxic and carcinogenic risks posed by PAHs in soils at a coking plant in Tangshan city. The toxic equivalent concentrations were relatively high in the boiler, crude benzol, and coal blending areas^15^. Therefore, ecological risk assessment according to 16 EPA priority PAHs can provide a support for petroleum pollutants control in different oilfields.

The principal objectives of this article were as follows: (1) to investigate and compare the distribution of PHs in three typical geographic oilfields soils in China, including the concentration level and occurrence characteristic of PHs; (2) to carry out fingerprint analysis on the contamination source profiles and the degradable potential of PHs based on the geochemical indices of *n*-alkanes and PAHs; (3) to evaluate the ecological potential risks in different sampling sites of oilfield associated with toxic equivalent quantity of carcinogenic PAHs, and provide suggested regional pollution control strategies on this basis.

## Materials and methods

### Research sites

The Shengli Oilfield (SL), Nanyang Oilfield (NY), and Yanchang Oilfield (YC) are three typical large oilfields located in east, central and west of China, respectively. SL Oilfield is located in the Yellow River delta of Shandong province and it is the second largest oil production base in China. The amount of oil resources is estimated at 1.45 × 10^6^ million tons and the exploration area is 1.94 × 10^5^ km^2^. The oil exploitation area Gudao is an efficient ecological economic zone of Dongying city, in Shandong Province. Due to its unique geographical location and natural environment, this area is prevailingly contaminated by PHs^16^. The NY is located in Nanyang Basin of Henan province and its amount of oil resources is estimated at 3.40 × 10^2^ million tons and the exploration area is 9.58 × 10^4^ km^2^^17^. It has been explored for nearly 50 years from 1972. The YC Oilfield is located in the loess plateau region in the northern part of Shanxi province, and has a long history and is the first oil well explored in 1907 in China^18^. The crude oil of YC Oilfield, NY Oilfield and SL Oilfield belong to typical light weight oil, medium weight oil and heavy weight oil, respectively, with different density, API, acidity, and sulfur content (shown in Supporting Information, Table S1). The oil exploitation and the development of petroleum industry exacerbated the petroleum contamination on these oilfield soils to varying degrees.

### Sample collection

**Figure 1:**
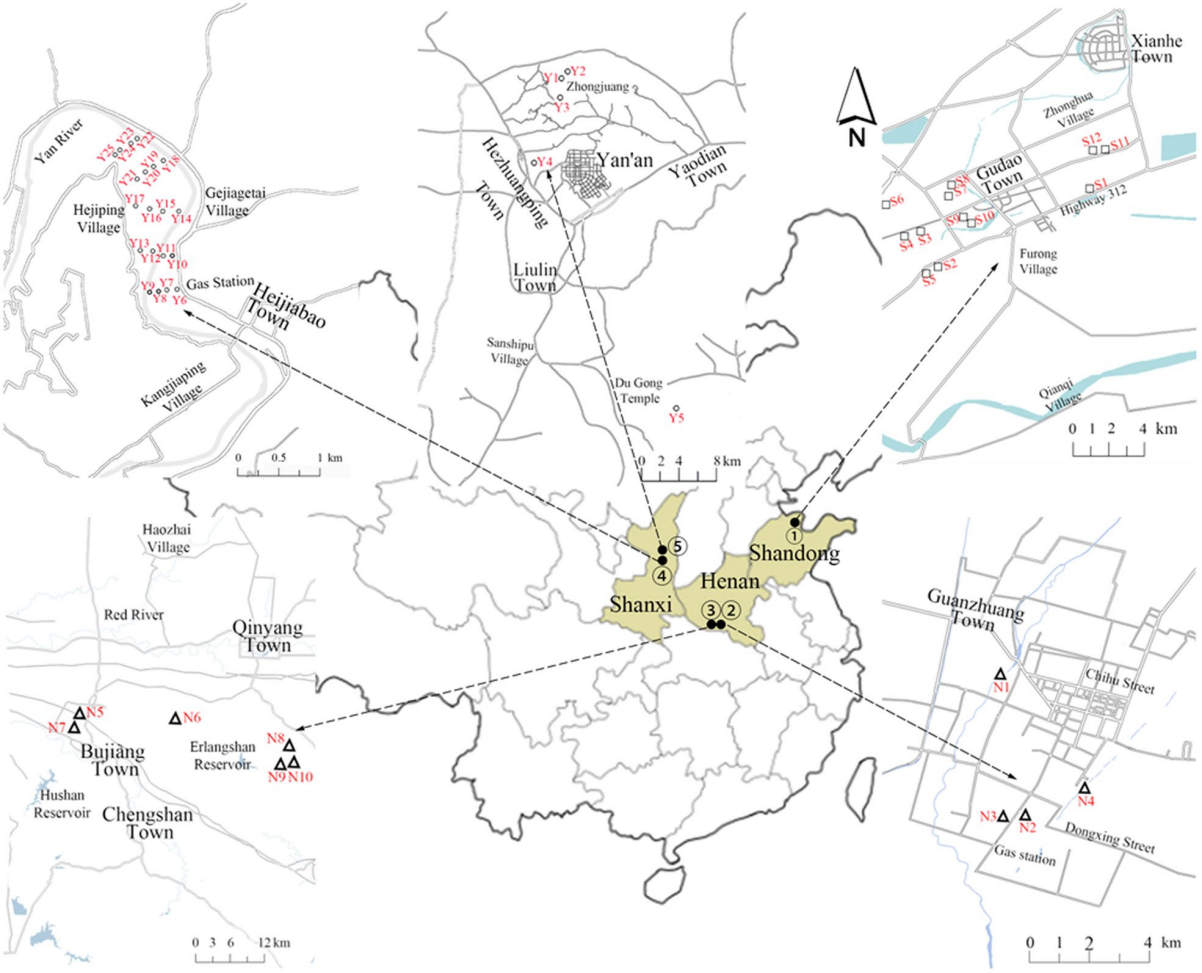
Locations of soil sampling points in three representative oilfields of China.

Surface soils (0–20 cm) were collected from the oilfield and its surrounding areas during September to December of 2019. The study areas were divided into five characteristic zones, viz. SL Oilfield (zone 1), NY Oilfield (zone 2 and 3) and YC Oilfield (zone 4 and 5) (Fig. 1). Total of 47 surface soils were collected from SL Oilfield (S1-S12), NY Oilfield (N1-N10), and YC Oilfield (Y1-Y25). To survey the regional pollution in oilfield, grid sampling method was applied in the collection of YC Oilfield (Y6-Y25), and the grid area was 800 m × 2000 m. Five subsamples were thoroughly mixed by coning and quartering to make a composite sample. Sampling soils were taken into brown bottles and transported in an icebox to the laboratory, and stored at 4 °C in laboratory till analysis. Description of sampling locations were recorded at the time of sampling (Supporting Information, Table S2). The geographic co-ordinates of sampling locations were recorded by GPS set (Garmin GPS Map 76CSX).

### Determination of oil components

#### Extraction and purification

Oil components were Soxhelt extracted, and then purified by using a silica gel-alumina column as described previously^19^. In brief, Five-gram aliquant of moist soil (after deduction of moisture content) was Soxhlet-extracted for 12 h with 120 mL dichloromethane at 54 °C^20^. Extracts were eluted from a glass column (dimensions: 20 mm × 400 mm) containing pre-rinsed activated silica gel and neutral aluminum (12 g:6 g, soaked with hexane) to purify the TPHs with addition of 100 mL dichloromethane, and using the following sequence of solvents: 20 mL of hexane, 70 mL of hexane and dichloromethane (1:1) to separate saturated hydrocarbons (SHs) and aromatic hydrocarbons (AHs). The separate components were concentrated to dryness by use of a rotary evaporator (Changcheng, Beijing, China), and then redissolve to 1 mL with hexane. The polar components were eluted by 100 mL dichloromethane and determined gravimetrically^21^. 100 µL aliquot of four surrogate standards (n-hexane-d14, n-undecane-d24, phenanthrene-d10, and benz[a]anthracene-d12) (each at a concentration of 2 µg/mL) were added to soil samples before extraction to estimate the extraction efficiency of *n*-alkanes and 16 PAHs, respectively.

#### Measurement of petroleum component

The concentration of TPHs, SHs, and AHs were determined by Gas chromatography (GC) according to the determination standards of petroleum hydrocarbons (C10-C40) announced by National Environmental Protection Standards of the People's Republic of China (HJ 1021-2019)^22^. Mixtures of 16 target PAHs on the EPA priority pollutant list (Supporting Information, Fig. S1) and 33 target *n*-alkanes (C8–C40) were purchased from J&K Scientific, and used as standards for external determination of PAHs and alkanes, with model of Agilent 7890 gas chromatograph connected to a 5975 Agilent HP mass spectrometer (Agilent, CA, USA)^23^. Components of PHs were separated by a Thermo Trace GC Ultra system equipped with a Thermo DB-5MS capillary column (30 m × 0.25 mm, i.d. 0.25 μm film thickness; Thermo Scientific, Runcorn, UK), operating with helium (99.99% purity) as the carrier gas at a constant flow of 1.0 mL/min. For determination of alkane components, the GC oven temperature was held at 70 °C for 1 min, and then temperature was increased by 4 °C/min from 70 °C to 300 °C, and held for 5 min. Similarly, for determination of 16 PAHs, the GC oven temperature was programmed as follows: 60 °C for 3 min, followed by a temperature increase of 15 °C/min to 180 °C, and then by 6 °C/min to 300 °C, held for 5 min. The injections were both conducted in splitless mode, and injector and interface temperature were both set at 280 °C. The mass spectrometer was operated with the ion source at 220 °C with an ionization energy of 70 eV. MS acquisition were conducted in selected ion monitoring (SIM) and scanning mode with m/z range of 45–600. The identification and quantification ions of 16 PAHs and alkanes were identified by comparing the retention time of calibration standard solution containing the target of each compound.

#### Quality assurance and control

To ensure the accuracy and reliability of the experimental analysis data, blank, substrates, and the parallel samples were analyzed. Each batch of samples extracted contained a method blank, which identified the external contamination occurred during the sample extraction and cleanup processes. Solvent blanks were added in each set of samples to test for carryover and background contamination. Standard samples with a specific concentration were added to each of the 10 samples used for analysis to recalibrate the retention time and peak area of the compound to ensure qualitative and quantitative accuracy. The PHs concentrations in the solvent and method blanks were all lower than the limit of quantification (LOQ), which was defined as the concentration of target peak five times the signal of solvent blank chromatograms. The method detection limit (MDL) was defined as the concentration giving a signal-to-noise ratio of 3 in the chromatograms of blank sample. The MDL of TPHs (C10-C40), SHs, AHs were both 1.0 mg/kg, the MDL of *n*-alkanes and 16 PAHs were 0.50–1.21 μg/kg and 0.67–1.68 μg/kg. The mean recoveries of the surrogate standards of *n*-alkanes and 16 PAHs were 87.1–112.4%, and 82.3–114.2%, respectively (Supporting Information, Table S3).

### Ecological risk assessment of PAHs

The toxic equivalent quantity (TEQ) was used to assess the ecological risk derived from PAHs, and toxic equivalent factors (TEFs) were introduced to assess the risk of PAH contribution^24^. In this study, due to great divergence of the sampling environment, we classified the soil site into three categories: petro-related area soils, farmland soils, and grassland soils. BaP was conducted to the standard reference and the TEQBaP values of the PAHs were calculated by the equation: $TEQBaP={\sum }_{i}^{n}{C}_{i}\cdot {TEF}_{i}$. Where *i* is the i-th PAH, n is the PAHs participating in the cumulative calculation, *Ci* is the concentration of the i-th PAHs (μg/kg), *TEFi* is the toxic equivalent factor of the i-th PAH, *TEQBaP* is the toxic equivalent quantity based on BAP (μg/kg). Comparison was made between *TEQBaP7* which were calculated based on seven kinds of carcinogenic PAHs (namely BaA, Chr, BbF, BkF, BaP, InP, DBA), and *TEQBaP16* which were calculated based on the 16 PAHs.

## Result and discussion

### Concentration of TPHs in surface soils

**Figure 2:**
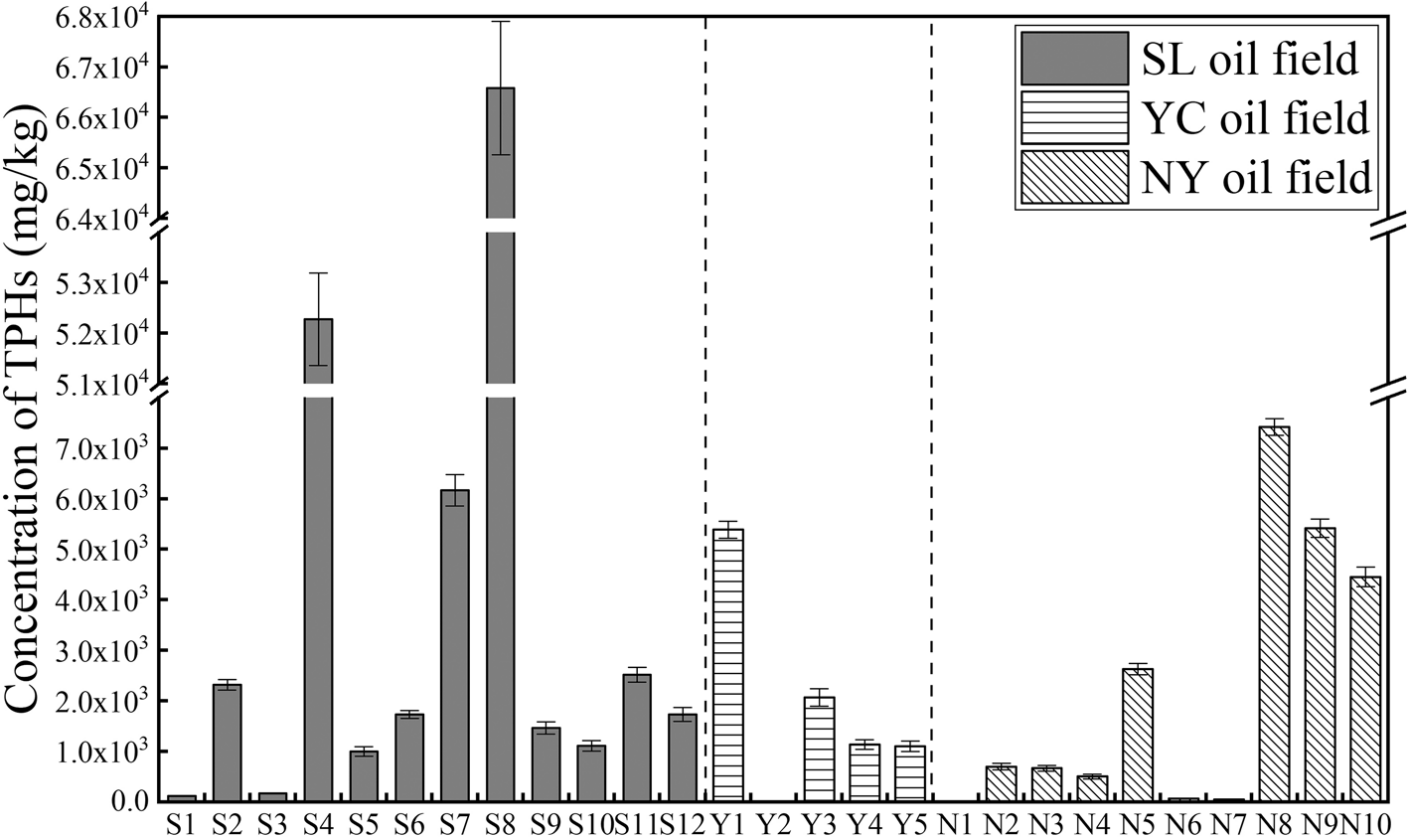
The concentration of TPHs in three oilfield soils.

**Figure 3:**
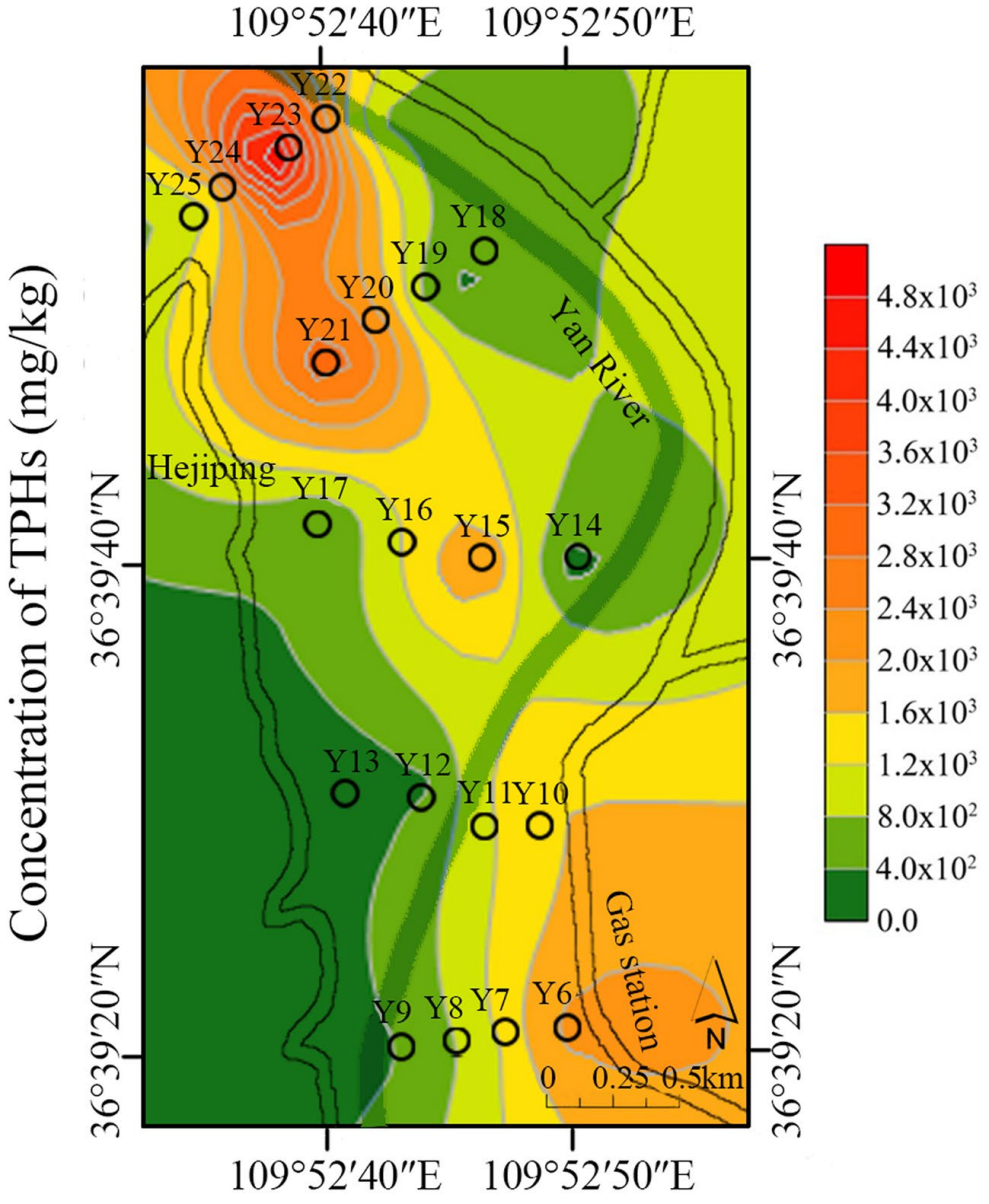
Grid regional distribution of TPHs in YC Oilfield.

Statistical results of TPHs concentrations at different geographic oilfields were showed in Fig. 2, and grid regional distribution of TPHs in YC Oilfield surface soils (Y6–Y25) were shown in Fig. 3. Results are given as mean value of triplicate analysis of each sample. The results of TPHs concentration in soil samples showed that the three oilfields all suffered from varying degrees of petroleum pollution, and 60.92% of the 47 sampling points was significantly higher than the soil critical value (500 mg/kg). The average concentration of the TPHs in each study areas conformed to be in the following law: SL Oilfield (average: 5.36 × 10^3^ mg/kg) $>$ NY Oilfield (average: 1.73 × 10^3^ mg/kg) $>$ YC Oilfield (average: 1.37 × 10^3^ mg/kg). The highest concentration of the TPHs were found in SL Oilfield surface soils, ranging from 1.21 × 10^2^ to 6.66 × 10^4^ mg/kg, and NY Oilfield had the second highest TPHs concentrations in the range from 15.82 to 7.42 × 10^3^ mg/kg. The concentrations of TPHs in YC Oilfield ranged from 12.34 to 5.38 × 10^3^ mg/kg. The petroleum contamination mainly derived from abandoned and working oil wells. S4 and S8 soils were collected near the abandoned oil well and working oil well, respectively, and had the highest concentration of TPHs up to 5.28 × 10^4^ and 6.66 × 10^4^ mg/kg. Y1, N8 near the abandoned oil well also had high concentration of TPHs with 5.39 × 10^3^ and 7.42 × 10^3^ mg/kg, respectively. Pollution caused by grounded crude oil in exploitation process has been a serious problem in oilfield area. Our previous research reported that the TPHs content in Dagang Oilfield soils collected adjacent to working oil wells were about 20-folds higher than that in corn soils and living area soils^25^. Concentration contour map of TPHs in YC Oilfield by grid sampling method showed that regional pollution in the northwest and southeast area are more serious than other sites. Y6 near the gas station and Y15, Y21, Y23 adjacent to the working oil wells have higher concentration (2.12 × 10^3^–5.34 × 10^3^ mg/kg) of TPHs than other farmland and grass soils. Previous study reported that the concentrations of TPHs ranged 7.0 × 10^2^–4.0 × 10^3^ mg/kg in oil exploitation areas of the loess plateau region (34°20′N,107°10′E), showing a similar pollution level with this study^26^.

The percentage composition of total PAHs, SHs and polar components of petroleum hydrocarbons were shown in Table 1. In general, the dominant petroleum component was saturated hydrocarbons in all soils, accounting more than 50%. Yet, the percentage proportion of PAHs and SHs in contamination soils adjacent to working and abandon oil wells were significantly different (*p* < 0.05). In detail, the proportion level of SHs in soils adjacent to working oil wells were the highest, accounting for 70.2–75.9%. The percentage proportion of SHs in soils near the abandoned oil wells accounted for 52.2–61.1%, decreasing with abandoned time of oil well lengthened. In contrast, the proportions level of PAHs in soils near the abandoned oil wells accounted the highest proportion for 30.4–34.4%. The proportions of petroleum hydrocarbons can indicate the biodegradation and natural aging process. Many researchers identified that SHs were more easily degraded by the indigenous microbes, and followed by PAHs, especially with more numbers of benzene rings. Polar components (resins and asphaltenes) are more intractable to metabolize compared with saturate and aromatic hydrocarbons^27^. It is confirmed by this study that the SHs were degraded initially and the PAHs and polar components accumulated in soils adjacent to abandoned wells. This study is aligned with Wang’s research that saturated hydrocarbon fraction gradually decreased, and aromatic fractions as well as polar components significantly accumulated over the duration of oil well use from the year of 1987–2014^21^. Devi et al. also confirmed that the higher concentration aromatic hydrocarbons frequently presented in the soils near the abandoned oil well than other five sampling sites surrounded by the group gathering stations in Sivasagar district of Assam, India^28^. There are more than 20 million of abandoned oil wells all over the world, so the investigation of contamination level of aromatic hydrocarbons and other toxic pollutants are benefit to abandoned oil wells for remediation and reutilization such as geothermal power generation^29,30^.

**Table 1:** The proportions of total PAHs, SHs and polar components.

Oilfield	Sample sites	Percentage composition (%)		
		PAHs	SHs	Polar components
SL oilfield	S1	23.1	55.3	21.6
	S2	24.5	58.7	16.8
	S3	26.8	60.1	13.1
	S4	30.4	62.2	7.4
	S5	23.6	59.5	16.9
	S6	22.4	58.3	19.3
	S7	25.2	68.5	6.3
	S8	24.7	69.4	5.9
	S9	25.1	72.2	2.7
	S10	23.3	71.4	5.3
	S11	22.2	75.9	1.9
	S12	23.6	72.3	4.1
NY oilfield	N1	23.1	66.7	10.2
	N2	22.2	75.3	2.5
	N3	23.6	71.6	4.8
	N4	34.7	54.9	10.4
	N5	22.5	72.4	5.1
	N6	23.5	67.8	8.7
	N7	25.1	64.2	10.7
	N8	34.1	56.8	9.1
	N9	25.5	72.1	2.4
	N10	23.2	70.2	6.6
YC oilfield	Y1	34.4	56.1	9.5
	Y2	20.6	50.2	29.2
	Y3	25.2	72.5	2.3
	Y4	26.3	56.8	16.9
	Y5	31.5	60.4	8.1
	Y6	23.3	74.2	2.5
	Y7	23.1	59.6	17.3
	Y8	24.4	57	18.6
	Y9	23.6	60.7	15.7
	Y10	22.5	56.6	20.9
	Y11	26.8	56.3	16.9
	Y12	23.1	60.2	16.7
	Y13	24.6	58.3	17.1
	Y14	22.4	59.1	18.5
	Y15	23.4	72.2	4.4
	Y16	23.7	52.2	24.1
	Y17	25.8	51.4	22.8
	Y18	24.6	50.6	24.8
	Y19	23.8	51.7	24.5
	Y20	23.1	58.4	18.5
	Y21	30.5	61.1	8.4
	Y22	23.5	53.2	23.3
	Y23	30.8	60.6	8.6
	Y24	25.3	52.1	22.6
	Y25	28.1	56.2	15.7

### Fingerprint characteristics of the geochemical indices of *n*-alkanes

**Figure 4:**
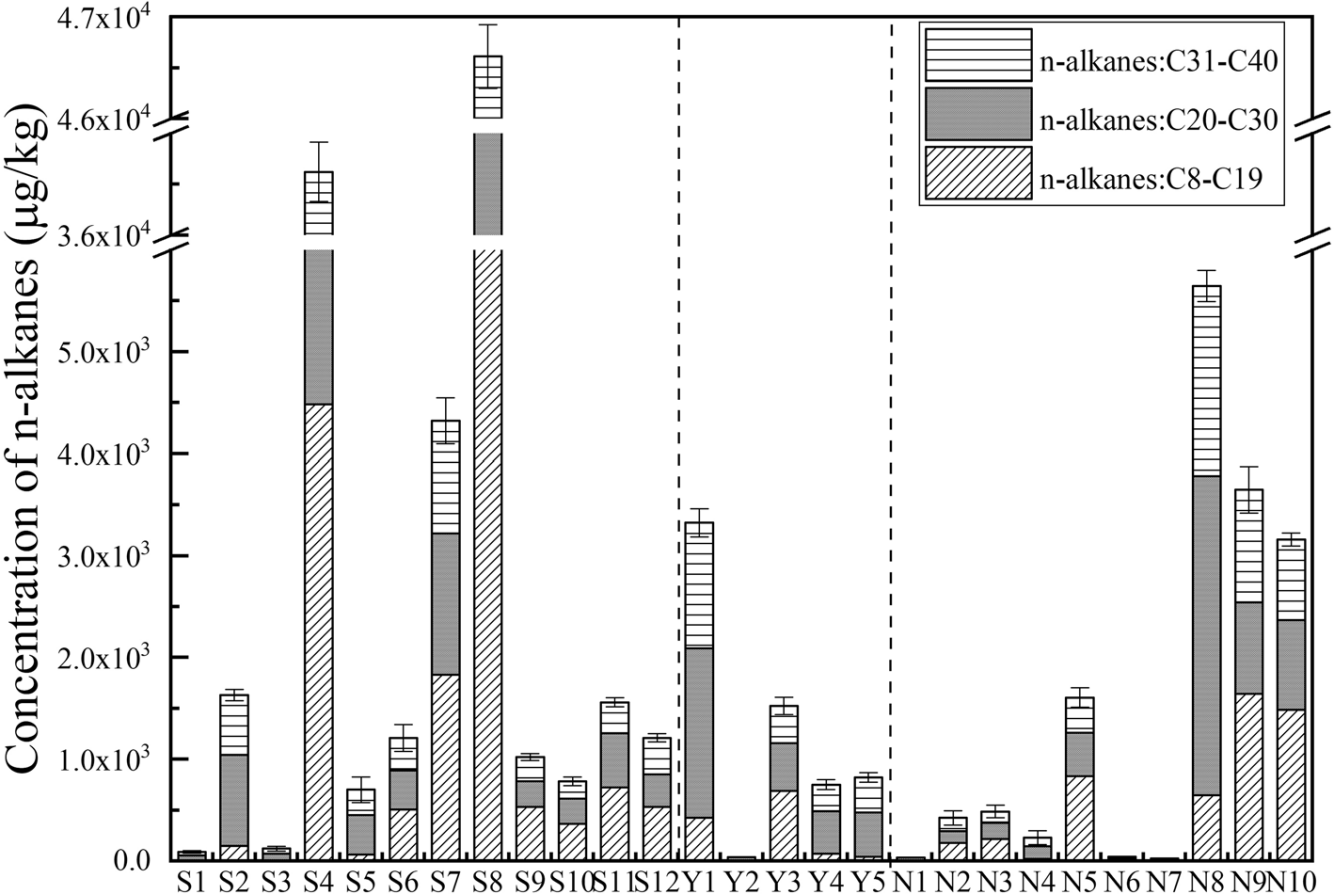
The concentration of *n*-alkanes in three oilfield soils.

The concentration of *n*-alkanes in three different geographic oilfield soils were showed in Fig. 4 and Supporting Information, Table S4. The *n*-alkanes were divided into three groups according to the length of carbon chain, i.e. short chain alkanes (C8–C19), medium chain alkanes (C20–C30), and long chain alkanes (C31–C40). The concentration of total *n*-alkanes showed a great difference in three oilfield soils (*p* < 0.05). Generally, the concentration of *n*-alkanes showed a similar law with the residual of TPHs. S8 and S4, Y1 and Y23, N8, N9, and N10 collected from oilfield exploitation areas had the higher concentration of *n*-alkanes than other soils in different Oilfields. It was noted that the *n*-alkanes content in grassland soils of Y10, Y20, and Y24 were higher than other grassland and farmland soils in YC Oilfield. Previous studies identified that partial grassland and farmland soils were also polluted by petroleum hydrocarbons, such like Sfax and Usinsk Oilfields^31,32^.

The composition of *n*-alkanes with low molecular weight (LMW) and high molecular weight (HMW) was the important indicators for petroleum migration and weathering process, and high ratio of LMW/ HMW illustrated a new petroleum contaminant input^33^. In this study, the proportions of the LMW *n*-alkanes (C8–C19) were more than 45% in all the soils adjacent to the working oil wells in contrast to soils adjacent to the abandoned wells of less than 20%. The HMW *n*-alkanes containing medium chain alkanes (C20–C30) and long chain alkanes (C31–C40) accumulated in the abandoned wells, and the short chain alkanes (C8–C19) has been biodegraded or vapored to some extent. This result was aligned with Wang’s study who identified the lower ratio of LMW/ HMW (less than 1) in abandoned well site than working well in Yellow River Delta^34^. This conclusion was also further proved by the geochemical indices of *n*-alkanes in this study.

The geochemical indices of *n*-alkanes were shown in Table 2. The geochemical indices of *n*-alkanes are used to fingerprint analysis of the oil contamination level, biogenic or petrogenic sources of petroleum, and the biodegradation capability^35^. The value of ∑n-alk/n-C16 of all the soils collected near to the abandoned and working oil wells were less than 30, indicating the oil exploitation was the dominant pollutant source in these sites. The value of ∑n-alk/n-C16 in petroleum processing plant (S2), gas station (S5, Y4), and oil transport area (N6) ranged from 30 to 50, demonstrating a contamination potential in the petro-related industries. As such, the petroleum contamination in the soils (except Y10, Y20, Y24) far away from oil wells was optimistic, where the value of ∑n-alk/n-C16 were more than 50. The value of ∑n-alk/n-C16 in Y10, Y20, Y24 were less than 30, revealing these sites were contaminated by petroleum hydrocarbons. These sites were located in the drainage basin of Yanhe River, this might be the result of petroleum migration caused by the rainfall runoff in the partial area^36^. Carbon preference index (CPI) is calculated by the ratio of odd to even carbon-numbered *n*-alkanes. The CPI analysis make clear the absence/presence of an anthropogenic inputs of petroleum hydrocarbons, of which values close to 1 indicate petroleum inputs, and higher CPI values (mostly between 3 and 6) indicate that the hydrocarbon components originated from vascular plant epicuticular wax^37^. In this study, CPI values in the contaminated soils ranged in 1–3, this confirmed that dominance petrogenic hydrocarbons and minor biogenic hydrocarbons were the oil pollutant source. CPI values of soils adjacent to working oil wells were close to 1, illustrating that the new petroleum input occurred. Similar conclusion was drew in previous study that the CPI values of surface soils near the working oil well were close to 1, whereas the control uncontaminated soils had the higher CPI values^35^. The dominant hydrocarbons derived from vascular plant epicuticular wax in most farmland and grassland soils, as CPI values ranged in 3–6.

**Table 2:** The geochemical indices of n-alkanes in different oilfield soils.

Samples	∑n-alk/n-C16	CPI	n-C17/Pr	n-C18/Ph	Pr/Ph
S1	60.07	4.29	nd	nd	nd
S2	41.31	3.54	1.64	0.95	0.74
S3	42.42	2.27	0.88	0.73	1.16
S4	28.02	1.92	0.93	0.95	0.99
S5	30.62	3.03	2.24	1.04	0.61
S6	48.36	3.88	1.67	1.23	2.34
S7	27.14	1.19	2.48	2.94	1.21
S8	25.58	1.29	2.54	2.96	1.18
S9	28.36	1.08	2.64	2.21	1.05
S10	26.6	1.15	2.33	2.07	1.13
S11	25.85	1.31	2.88	2.96	1.22
S12	24.03	1.24	2.41	2.05	1.14
N1	67.24	4.25	nd	nd	nd
N2	22.24	1.03	2.42	2.37	1.28
N3	23.65	1.27	2.34	3.23	1.12
N4	28.92	2.32	0.82	0.53	0.86
N5	22.57	1.44	2.37	2.03	1.17
N6	46.23	3.48	nd	nd	nd
N7	60.21	4.72	nd	nd	nd
N8	29.13	1.24	0.82	0.72	0.68
N9	25.58	1.18	2.73	2.35	1.03
N10	23.29	1.32	2.94	2.83	1.16
Y1	26.17	1.23	0.92	0.82	0.83
Y2	60.32	4.82	nd	nd	nd
Y3	25.21	1.57	2.83	2.34	1.23
Y4	30.13	2.32	1.82	1.24	1.31
Y5	28.25	1.98	0.67	0.53	0.94
Y6	23.33	1.72	2.46	2.39	1.15
Y7	52.15	4.98	nd	nd	nd
Y8	54.32	5.24	nd	nd	nd
Y9	57.42	3.98	nd	nd	nd
Y10	27.23	1.23	2.13	2.34	0.84
Y11	58.25	4.89	nd	nd	nd
Y12	60.14	4.38	nd	nd	nd
Y13	52.19	5.39	nd	nd	nd
Y14	57.45	4.62	nd	nd	nd
Y15	23.45	1.83	2.88	1.93	1.27
Y16	55.46	3.82	nd	nd	nd
Y17	61.31	4.72	nd	nd	nd
Y18	57.49	5.31	nd	nd	nd
Y19	55.42	4.92	nd	nd	nd
Y20	28.31	2.14	1.92	2.25	1.24
Y21	23.56	1.92	0.92	0.56	0.88
Y22	52.34	3.24	nd	nd	nd
Y23	24.16	1.88	0.76	0.57	0.95
Y24	28.32	2.16	2.12	2.82	1.58
Y25	57.23	4.55	nd	nd	nd

∑n-alk/n-C16: Sum of *n*-alkanes (C8-C40) over to *n*-alkane (C16); CPI: odd to even carbon preference index from n-C8 to n-C40; C17/Pr: ratio of n-alkane (C17) to pristane; C18/Ph: ratio of *n*-alkane (C18) to phytane; Pr/Ph: ratio of pristane to phytane; nd: not detected.

Pristane (Pr) and phytane (Ph) are the representative acyclic isoprenoid alkanes, and the ratios of n-C17/Pr, n-C18/Ph, Pr/Ph are usually used as indicator to assess the weathering and biodegradation extent of petroleum^35^. The acyclic isoprenoid alkanes were not detected in the most farmland and grassland soils without contamination by petroleum. The lower ratios of n-C17/Pr (0.76 to 0.93), n-C18/Ph (0.53 to 0.95), Pr/Ph (0.83 to 0.99) were in the soils adjacent to abandoned oil wells than adjacent to working oil wells, with ratios of n-C17/Pr (from 2.33 to 2.88), n-C18/Ph (from 1.93 to 2.96), Pr/Ph (from 1.03 to 1.27). It indicated that more hydrocarbons biodegradation occurred in the long-term oil contaminated soil than in soils that were freshly contaminated^38^.

### Concentration and source characters of PAHs

**Figure 5:**
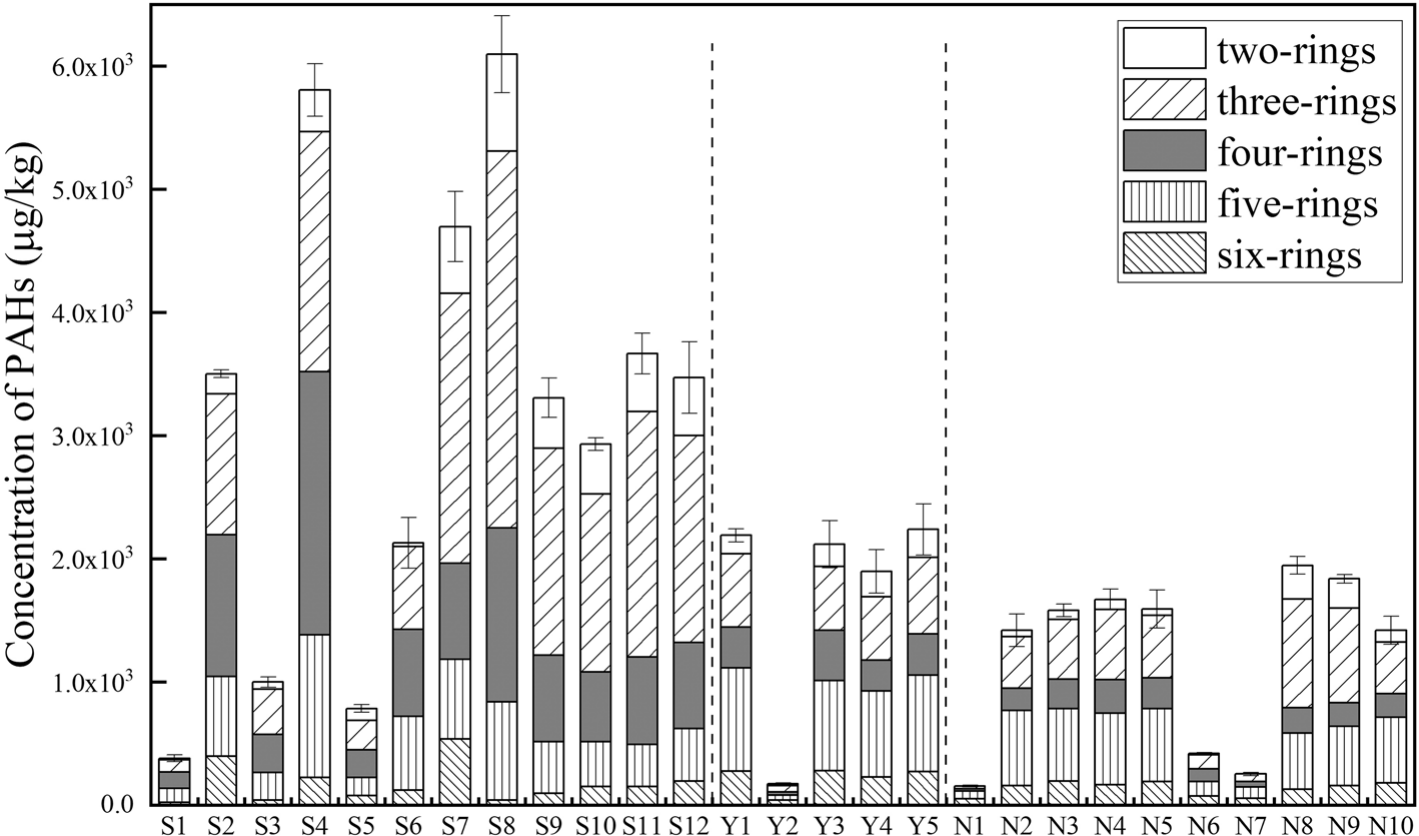
The concentration of PAHs in three different geographic oilfield soils.

The total concentration of the 16 ∑PAHs in three oilfield soils were shown in Fig. 5, and concentration of PAHs in YC Oilfield soils by grid sampling method were shown (Supporting Information, Table S5). Owing to the different sampling environment, 16 ∑PAHs content showed a great divergency in three oilfield soils, SL Oilfield ranging in 1.81 × 10^2^ μg/kg–6.09 × 10^3^ μg/kg, NY Oilfield ranging in 1.55 × 10^2^ μg/kg–1.94 × 10^3^ μg/kg, YC Oilfield ranging in 1.73 × 10^2^ μg/kg–2.24 × 10^3^ μg/kg. S4 adjacent to abandoned oil wells, S9-S12 adjacent to working oil wells, and S8 collected from oil sludge soil have the higher concentration of 16 ∑PAH than other SL soils. N8, N9 and Y1, Y5 have the highest 16 ∑PAHs content in NY and YC Oilfield soils, respectively. According to the previous study, soil contamination is categorized into four classes based on the concentration of ∑PAHs, ie: ∑PAHs concentration < 200 μg/kg, uncontaminated; ∑PAHs concentration ranging in 200–600 μg/kg, weakly contaminated; ∑PAHs concentration ranging in 600–1000 μg/kg, contaminated; and ∑PAHs concentration > 1000 μg/kg, heavily contaminated^39^. In this research, a heavily contaminated tendency was found in three oilfield soils, except the farmland and grassland with concentration of 16 ∑PAHs less than 200 μg/kg, where were less affected by the oil-related activities.

PAHs in the environment mainly derive from energy utilization and human activities, and the composition characteristics of PAHs can illustrate their pyrolytic and petrogenic source. Studies have shown that low-ring PAHs (two to three benzene rings) mainly derived from petroleum input, such as oil leakage and ground crude oil. High-ring PAHs (four to six benzene rings) mainly derived from combustion sources, including incomplete combustion of biomass like grass and straw, and fossil fuels (petroleum and coal)^40^. In this study, the proportions of the low-ring PAHs in three oilfields accounted for 29.69–67.14% (SL), 32.89–38.93% (YC), and 15.07–59.4% (NY), respectively. Especially, the proportions of low-ring PAHs were the highest in S4, S7–S12, N8, N9, Y1, Y5, which were collected near the working and abandoned oil wells. This indicated that the petrogenic PAHs were the dominant source in these areas. Soils collected from petrochemical-related sites, such as petroleum processing plant (S2), gas station (S5, Y4), transport area (N6), and farmland (S1) as well as grassland soils (Y1-Y14, Y22, and Y25) had higher proportion of high-ring PAHs component, with more than 53%, indicating the pyrolytic PAHs were the dominant source of aromatic components in these sampling sites.

**Figure 6:**
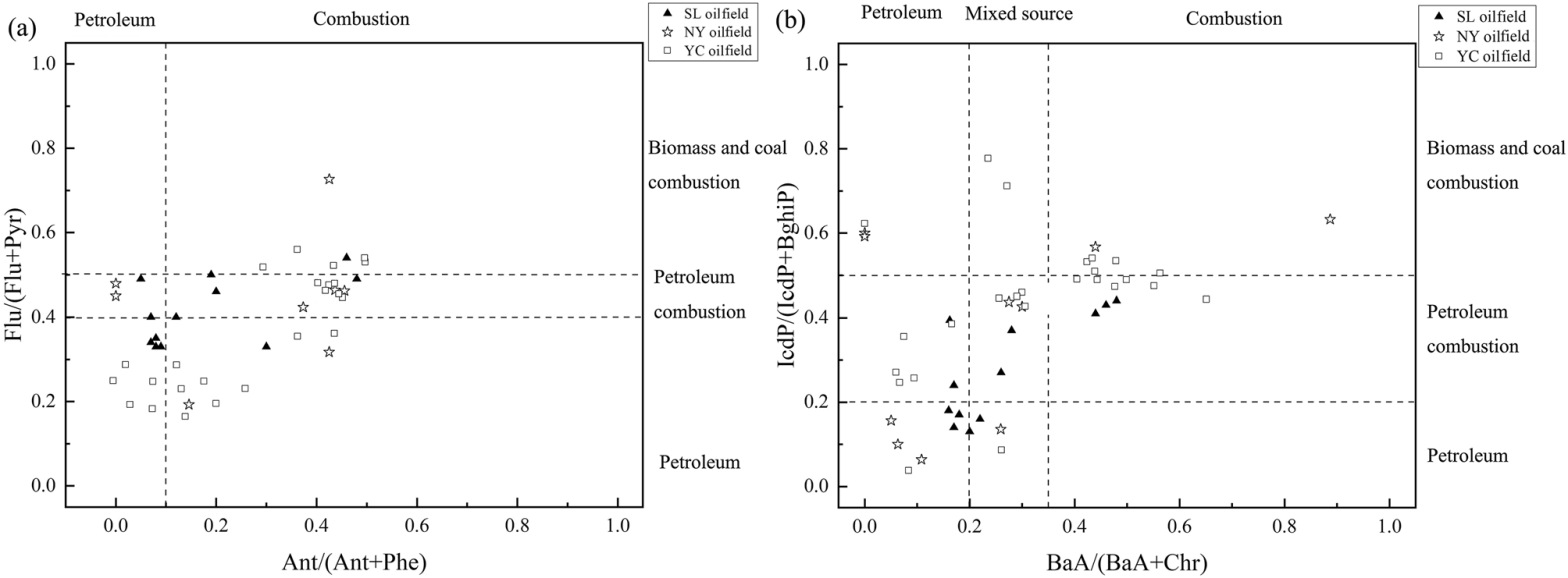
Diagnostic ratio for analysis in source of PAHs in three oilfield soils. (**a**) ratios plot of Flu/(Flu + Pyr) and Ant/ (Ant + Phe); (**b**) ratios plot of IcdP/(IcdP + BghiP) and BaA/(BaA + Chr).

The ratio of indeno [1,2,3-cd] pyrene to indeno [1,2,3-cd] pyrene plus benzo[g,h,i] perylene [IcdP/(IcdP + BghiP)], and benz[a]anthracene to benz[a]anthracene plus chrysene [BaA/(BaA + Chr)], fluoranthene to fluoranthene plus pyrene [Flu/(Flu + Pyr)], anthracene to anthracene plus phenanthrene [Ant/(Ant + Phe)] were used to clarify the possible sources of PAHs. Refer to previous research, a value ratio of Flu/(Flu + Pyr) ratio < 0.4 indicates that PAHs are derived from petroleum source, ratio between 0.4 and 0.5 indicates that from petroleum combustion and ratio > 0.5 indicates that from biomass and coal combustion. The Ant / (Ant + Phe) ratio less than 0.1 means that PAHs are derived from petroleum, otherwise they are derived from combustion source. A value ratio of IcdP/(IcdP + BghiP) ratio < 0.2 indicates that PAHs are mainly derived from petroleum source, ratio between 0.2 and 0.5 indicates that from petroleum combustion, whereas ratio > 0.5 indicates that from biomass and coal combustion. A value ratio of BaA/(BaA + Chr) < 0.2 indicates that PAHs are mainly derived from petroleum source, ratio between 0.2 and 3.5 indicates from a mixed source including petroleum and combustion, whereas ratio > 0.35 indicates from combustion source^41^. A ratios plot of Flu/(Flu + Pyr) and Ant/ (Ant + Phe) was shown in Fig. 6a, ratios of IcdP/(IcdP + BghiP) and BaA/(BaA + Chr) was shown in Fig. 6b. The source of PAHs in SL Oilfield mainly originates from the petroleum and petroleum combustion. This can be reasonable explained by that the most SL Oilfield soils were collected near the oil exploitation area and petroleum related industry, such as petroleum refining, oil transportation, and gas station. Therefore, upgrading oil exploitation equipment and improving transport efficiency are the critical control pathway for PAHs pollution caused by oil spilling and landing in SL Oilfield. The source of PAHs in NY and YC Oilfield were complex, due to varieties of sampling environment. The soils sampled nearby abandoned and working oil wells were the dominant petroleum source, whereas the mixed source (petroleum and petroleum combustion) made the greatest devotion to PAHs origination except in farmland and grassland soils far away the petro-related activities. Avoiding oil spills accident and limiting tailpipe emissions from transport vehicles are the effective PAHs source control method in petro-related areas of NY and YC Oilfield. Meanwhile, oil-blocking isolation zones should be set up to prevent PAHs pervasion to the surrounding grasslands and farmlands, especially in the lower reaches of the Yanhe River. The PAHs source of farmland and grassland (S1, N1, N7, Y2, Y11, Y12, Y14, Y22, Y25) mainly derived from biomass and coal combustion, illustrating that the heating in winter and biomass (wood, grass, and straw) combustion made a great contribution to the PAHs accumulation. Therefore, the most important management policies are to prohibit burning of wasteland and raise the awareness of environment-friendly heating among local people, as well as explore the way of biomass efficient utilization, such as turning straw into fertilizer and feed^42^, or producing bio-energy^43^. The regional industry has a great influence on the derivation of PAHs in this study, and the distribution of PAHs varies in different regions. Previous studies have descripted that dominant PAHs derived from anthropogenic sources, such as oil extraction, petroleum processing and refining, municipal waste incineration, automobile exhaust emissions, coal and biomass (wood, grass, and straw) burning^44^. Overall, the source characteristics of PAHs based on the isomer ratio and PAHs rings can prove a critical strategy on the source control of the PAHs contamination.

### Ecological risk assessment of PAHs

The occurrence characters of PAHs, especially derived from anthropogenic activities has raised great concerns over the ecological and health risk, due to their carcinogenic, teratogenic, and mutagenic properties^44^. The descriptive statistic TEQBap of PAHs in different sampling sites was shown in Table 3. The concentration of PAHs in Dutch Soil Standard was taken as the reference target values^45^. TEQs of ∑PAH16 in petro-related area soils were in the highest range of 620.93 μg/kg–1434.21 μg/kg, with a mean of 1149.97 μg/kg, whereas lowest TEQs of ∑PAH16 were present in farmland soils, ranging in 19.91–22.59 μg/kg, with a mean of 19.91 μg/kg. TEQs of ∑PAH16 in grassland soils were in the range of 28.64–426.84 μg/kg, with a mean of 373.47 μg/kg. Compared with TEQs of ∑PAH16, TEQs of ∑PAH7 (seven carcinogenic PAHs marked by *) were both closed to the value of ∑PAH16 in three categories, accounting for more than 90% percent of the ∑PAH16. It suggested that the seven carcinogenic PAHs were the dominant contributor to TEQs of ∑PAH16. The contribution of individual PAHs to the TEQs of ∑PAH16 showed similar regular in the following order: BaP (32.06–39.17%) > DBA (31.94–36.63%) > BbF (14.16–21.87%) ≫ BaA, Chr, InP, and BkF (less than 10%). This result aligned to the previous study that the contribution of individual PAHs to the TEQs of ∑PAH16 was BaP (45%) > DBA (33%) in urban surface dust of Xi’an city, China^46^. Therefore, contamination control should priority focus on the individual PAHs of BaP, DBA, BbF in these areas. In addition, the ecological risk with abandoned time ranging 0–15 years has been assessed, and the descriptive statistic TEQBap of PAHs was shown in Supporting Information, Table S6. The highest TEQs of ∑PAH16 and ∑PAH7 with mean of 1422.27 μg/kg and 1400.48 μg/kg, respectively, were present in soils adjacent to abandoned oil well with abandoned time of 0—5 years. And the TEQs of ∑PAH16 and ∑PAH7 decreased with the abandoned time though the percentage proportion of PAHs increased. The TEQs of ∑PAH16 and ∑PAH7 were close between abandoned time of 5–10 years and 10—15 years while both had high content. It demonstrated that high ecological risk was persistent in abandoned oil well areas over abandoned time of 15 years, and basically stable after 5 years. Therefore, abandoned oil well areas need to be blocked to prevent PAHs entering the external environment, and combine physical–chemical technology for petroleum remediation instead of simple weathering biological processes.

**Table 3:** Descriptive statistic TEQBap of PAHs in different sampling area.

PAHs	Dutch Soil Standard (μg/kg)	TEF	TEQBap of petro-related area soils (μg/kg)			TEQBap of grassland soils (μg/kg)			TEQBap of farmland soils (μg/kg)		
			Range	Mean	SD	Range	Mean	SD	Range	Mean	SD
Nap	15	0.001	0.24–0.54	0.42	0.02	0.006–0.14	0.13	0.02	0.0052–0.0093	0.0072	0.002
Acy	–	0.001	0.64–0.92	0.83	0.03	0.04–0.32	0.28	0.05	0.013–0.016	0.015	0.003
Ace	–	0.001	0.46–1.85	1.41	0.04	0.03–0.35	0.38	0.07	0.032–0.036	0.035	0.004
Flu	–	0.001	1.35–3.92	2.1	0.23	0.32–0.92	0.72	0.33	0.024–0.042	0.038	0.007
Phe	50	0.001	0.89–3.52	2.68	0.43	0.82–1.52	1.15	0.65	0.653–1.07	0.87	0.023
Ant	50	0.01	1.67–6.02	4.78	0.46	0.66–1.02	0.87	0.06	0.036–0.064	0.048	0.025
Fluo	15	0.001	0.05–1.25	0.18	0.012	0.09–1.23	0.15	0.043	0.053–0.095	0.088	0.037
Pyr	–	0.001	0.28–0.75	0.65	0.34	0.08–0.25	0.21	0.03	0.002–0.005	0.004	0.002
BaA*	20	0.1	44.27–78.99	68.64	12.63	4.23–18.23	15.32	2.73	0.64–1.24	1.08	0.23
Chr*	20	0.001	1.65–3.42	2.032	0.29	0.35–0.92	0.76	0.14	0.037–0.058	0.041	0.012
BbF*	–	0.1	126.04–283.33	251.36	23.47	4.02–83.74	72.3	13.32	1.85–3.45	2.82	0.93
BkF*	25	0.1	3.25–33.27	8.57	1.22	1.23–10.23	7.68	0.28	0.52–0.61	0.61	0.25
BaP*	25	1	119.44–466.39	368.48	35.87	9.24–166.39	146.14	25.44	6.52–8.42	7.76	1.26
InP*	25	0.1	10.27–538.73	16.78	2.18	0.24–3.23	2.12	0.18	0.13–0.16	0.13	0.024
DBA*	–	1	310.42–542.76	420.96	32.76	7.28–138.3	125.23	12.75	5.21–7.31	6.36	2.98
BghiP	20	0.01	0.01–1.03	0.096	0.034	0.01–0.05	0.03	0.002	0.0008–0.0021	0.0012	0.0008
∑PAH16	32.8	–	620.93–1434.21	1149.97	110.02	28.64–426.84	373.47	56.095	19.91–22.59	19.91	5.7898
∑PAH7	32.02	–	615.35–1415.44	1136.82	108.42	26.24–418.92	369.55	54.84	18.81–21.25	18.8	5.686

*Indicate carcinogenic PAHs; *TEQBap*: toxic equivalent quantity based on BaP; *∑PAH16*: sum of 16 converted PAH concentrations based on toxic equivalents of BaP. *∑PAH7*: sum of seven carcinogenic PAHs concentrations based on toxic equivalents of BaP; *TEF*: toxic equivalency factor; *SD*: standard deviation.

As referred the PAHs standard of Dutch soil, TEQs of ∑PAH7 was 32.02 μg/kg, calculated by ten individual PAHs times TEFs. In this study, the mean TEQs of ∑PAH7 were about 35- and 10-folds of Dutch soil in petro-related area soils and grassland soils, indicating a high and medium ecological risk in these soils respectively. However, the mean TEQs of ∑PAH7 in farmland soils (18.80 μg/kg) was below Dutch soil, presenting a low potential ecological risk. It should be noted that the minimum of TEQs of ∑PAH7 in grassland soil was 26.24 μg/kg less than TEQs of ∑PAH7 in Dutch soil, but it was vulnerable affected by the surrounding soils with high TEQs of ∑PAH7. In this study, except the farmland soils, TEQs of ∑PAH7 exhibited higher TEQ values than those reported soils in Santiago, Chile^47^ and Nepal^24^, and road dust in Tianjin, China^48^. Overall, the most threat of ecological risk in petro-related soils caused by the anthropogenic PAHs input, such like oil leakage, oil refining, and fossil energy combustion. Preventing oil spills accident and developing the remediation methods are the main significant ways to reduce the ecological risks in these areas. The medium ecological risk in grassland might result from the migration of PAHs via rainfall pathway. Therefore, establishment the oil-blocking isolation zones is the critical way for medium ecological risk areas to control petroleum inflow. Even though the low ecological risk was identified in farmland soils, PAHs source analysis indicated that the biomass combustion should be controlled in these areas.

## Conclusion

The results of this study revealed the occurrence characteristics and source of petroleum hydrocarbons in three different geographical oilfields soils of China by fingerprint analysis. Moreover, ecological potential risk of PAHs was assessed according to the toxic equivalent quantity of carcinogenic PAHs. The main conclusions are as follows: (1) The general oil contamination level was higher in SL Oilfield soils than other NY and YC Oilfield, and the higher concentration of TPHs were found in soils adjacent to the working and abandoned oil wells as well as partial grassland collected from YC Oilfield; (2) The higher proportion of LMW *n*-alkanes over total *n*-alkanes in soils adjacent to the working oil wells indicated new petroleum contaminant input occurred, whereas the saturated hydrocarbons were largely degraded in soils adjacent to the abandoned wells, confirmed by lower proportion of LMW *n*-alkanes and lower ratio of *n*-alkanes to acyclic isoprenoid alkanes; (3) The PAHs of oil exploitation areas mainly derived from petroleum source in three oilfield soils, however the PAHs of soils collected from petrochemical-related sites, such as petroleum processing plant, gas station, and transport areas mainly derived from the petroleum combustion. The biomass combustion was the dominant PAHs source in farmland and grassland soil far away from the oil exploitation activities. The suggestive petroleum control strategies were proposed, such as upgrading oil exploitation equipment in oil exploitation, setting up oil-blocking isolation zones in abandoned oil well areas and drainage basin of Yanhe River, and prohibiting burning of wasteland in farmland and grassland; (4) High, medium, and low ecological risk from carcinogenic PAHs presented in petro-related area soils, grassland soils, and farmland soils, respectively. High ecological risk was persistent in abandoned oil well areas over abandoned time of 15 years, and basically stable after 5 years.

## Supplementary Information

Supplementary Information.

## Data Availability

The authors declare that all data supporting the findings of this study are available within the article and its supplementary information files.
