# Supplementary material for: Fingerprint analysis reveals sources of petroleum hydrocarbons in soils of different geographical oilfields of China and its ecological assessment
Source: Sci Rep. 2022 Mar 21;12:4808. doi: 10.1038/s41598-022-08906-6 (PMC8938453; doi:10.1038/s41598-022-08906-6)
Supplement: Supplementary file 1 — Supplementary Information. [file 41598_2022_8906_MOESM1_ESM.pdf]

Scientific Report

Supplementary Material

Fingerprint analysis reveals sources of petroleum hydrocarbons in soils of different geographical oilfields of China and its ecological assessment

Qinglong Liu[1,2], Chunqing Xia[1], Lan Wang[1,2,3*], Jingchun Tang[1,2,3]*

1. College of Environmental Science and Engineering, Nankai University, Tianjin 300071, China

2. Tianjin Engineering Center of Environmental Diagnosis and Contamination Remediation, Tianjin 300071, China

3. Key Laboratory of Pollution Processes and Environmental Criteria (Ministry of Education), Tianjin 300071, China.

* Corresponding author: College of Environmental Science and Engineering, Nankai University, 38 Tongyan Road, Jinnan District, Tianjin 300350, China

E-mail address: tangjch@nankai.edu.cn (J. Tang), envwangl@nankai.edu.cn (L. Wang)

Supporting Information

Table S1 Properties of crude oil from three different oilfields

Table S2 Sample sites and soil types in three geographic oilfields

Table S3 Results of the recoveries of surrogate standards

Table S4 The concentration of n-alkanes in YC oilfield soils by grid sampling

Table S5 The concentration of PAHs in YC oilfield soils by grid sampling

Table S6 Descriptive statistic TEQBap of PAHs in abandoned oil well area

Fig. S1 Structures and nomenclatures of the 16 PAHs on the EPA priority pollutant list

Table S1 Properties of crude oil from three different oilfields

Oilfield	Density (g/cm^3)	API	Acidity (mg KOH/g)	Sulfur content (w%)	Classification
YC Oilfield	0.8404	36.1	0.06	0.07	light weight oil
NY Oilfield	0.8761	29.3	0.6	0.11	medium weight oil
SL Oilfield	0.9079	23.8	0.98	0.85	heavy weight oil

Table S2 Sample sites and soil types in three geographic oilfields.

Soil numbers	Samples sites	Latitude	Longitude
S1	paddy soil	37°51′55″	118°49′90″
S2	petroleum processing plant	37°49′40″	118°45′50″
S3	pipeline	37°50′46″	118°45′22″
S4	abandoned oil well, abandoned for 3 years	37°50′36″	118°44′50″
S5	gas station	37°49′28″	118°45′32″
S6	soil remediation plant	37°51′25″	118°44′20″
S7	1# oil sludge	37°51′40″	118°46′15″
S8	2# oil sludge	37°51′60″	118°46′20″
S9	1# working oil well，R=5m	37°51′10″	118°46′36″
S10	1# working oil well，R=10m	37°50′57″	118°46′50″
S11	2# working oil well，R=5m	37°52′58″	118°50′59″
S12	2# working oil well，R=10m	37°52′25″	118°52′80″
N1	farmland soil	32°41′51″	112°35′26″
N2	1# working oil well	32°39′19″	112°35′46″
N3	2# working oil well	32°39′16″	112°35′43″
N4	1# abandoned oil well, abandoned for 11 years	32°39′44″	112°36′57″
N5	3# working oil well	32°36′56″	113°0′58″
N6	Transport area	32°35′59″	113°0′58″
N7	farmland soil	32°33′44″	113°2′46″
N8	2# abandoned oil well, R=2m, abandoned for 13 years	32°36′3″	113°1′0″
N9	4# working oil well, R=2m	32°33′31″	113°2′54″
N10	4# working oil well, R=5m	32°33′31″	113°2′54″
Y1	1#abandoned oil well, R=2m, abandoned for 15 years	36°42′49″	109°28′3″
Y2	farmland soil	36°42′50″	109°28′3″
Y3	1#working oil well	36°42′1″	109°28′2″
Y4	gas station	36°39′25″	109°26′5″
Y5	2#abandoned oil well, abandoned for 5 years	36°39′56″	109°52′51″
Y6	2#working oil well	36°39′21″	109°52′53″
Y7	grassland soil	36°39′19″	109°52′51″
Y8	grassland soil	36°39′17″	109°52′49″
Y9	grassland soil	36°39′31″	109°52′50″
Y10	grassland soil	36°39′31″	109°52′47″
Y11	grassland soil	36°39′32″	109°52′43″
Y12	grassland soil	36°39′45″	109°52′52″
Y13	grassland soil	36°39′45″	109°52′50″
Y14	grassland soil	36°39′45″	109°52′48″
Y15	3#working oil well	36°39′54″	109°52′48″
Y16	grassland soil	36°39′51″	109°52′45″
Y17	grassland soil	36°39′49″	109°52′41″
Y18	grassland soil	36°40′0″	109°52′36″
Y19	grassland soil	36°39′59″	109°52′35″
Y20	grassland soil	36°39′59″	109°52′33″
Y21	3#abandoned oil well, abandoned for 8 years	36°39′52″	109°52′50″
Y22	grassland soil	36°40′01″	109°52′36″
Y23	4#abandoned oil well, abandoned for 10 years	36°39′59″	109°52′35″
Y24	grassland soil	36°39′59″	109°52′33″
Y25	grassland soil	36°39′56″	109°52′30″

Table S3 Results of the recoveries of surrogate standards

Soil numbers	n-hexane-d14 Recovery (%)	n-undecane-d24 Recovery (%)	Average recovery (%)	phenanthrene-d10 Recovery (%)	benz[a]anthracene-d12 Recovery (%)	Average recovery (%)
S1	99.1	100.4	99.75	85.4	101.05	93.2
S2	101.3	99.6	100.4	92	93.2	92.6
S3	89.5	95.2	92.3	93.8	94.85	94.3
S4	114.4	104.6	109.5	85.5	111.1	98.3
S5	90.6	110.5	100.5	106.7	109.05	107.9
S6	98.8	98.2	98.5	117.1	105.85	111.5
S7	92.5	96.7	94.6	106.7	105.7	106.2
S8	101.1	111.4	106.2	91.5	87.1	89.3
S9	85.8	110.7	98.2	101.6	94.7	98.2
S10	91.9	121.3	106.6	108.9	106.9	107.9
S11	98.1	118.2	108.1	98.55	98.8	98.7
S12	85.1	89.1	87.1	106.9	99.2	103.1
N1	91.7	111.4	101.5	108.45	96.9	102.7
N2	93.5	93.9	93.7	87.4	98	92.7
N3	85.2	105.5	95.3	84.7	79.9	82.3
N4	106.4	116.8	111.6	96.75	111.9	104.3
N5	112.7	106.4	109.5	107.25	95.25	101.3
N6	121.5	91.2	106.3	107.5	120.9	114.2
N7	111.1	101.3	106.2	98.5	101.6	100.1
N8	98.3	108.6	103.4	112.5	87.3	99.9
N9	87.6	101.4	94.5	92.1	92.3	92.2
N10	95.2	105.5	100.3	93.8	98.1	96
Y1	107.4	107.7	107.5	106	89.65	97.8
Y2	99.3	85.6	92.4	99.8	94.3	97.1
Y3	101.1	91.7	96.4	110.2	99.75	105
Y4	99.4	93.5	96.5	100	97.8	98.9
Y5	114.5	99.4	106.9	121.8	85.1	103.5
Y6	91.3	121.4	106.3	111.4	91.2	101.3
Y7	98.2	99.7	98.9	98.6	93	95.8
Y8	112.2	112.6	112.4	107.7	98.9	103.3
Y9	91.8	99.7	95.7	99.6	120.9	110.3
Y10	93.5	97.4	95.4	101.4	99.2	100.3
Y11	105.7	98.5	102.1	99.7	93.2	96.5
Y12	96.2	79.4	87.8	114.8	94.85	104.8
Y13	86.1	99.5	92.8	91.6	111.1	101.4
Y14	87.3	109.9	98.6	112.7	109.05	110.9
Y15	92.1	99.7	95.9	96.05	105.85	101
Y16	93.5	121.5	107.5	95.75	110.6	103.2
Y17	101.3	111.1	106.2	102.4	97.8	100.1
Y18	104.5	98.3	101.4	88.1	106.9	97.5
Y19	91.8	87.6	89.7	93.1	98.8	96
Y20	89.7	98.2	93.95	90.45	98.1	94.3
Y21	97.4	112.2	104.8	95.1	95.4	95.3
Y22	88.5	91.8	90.2	100.55	107	103.8
Y23	96.1	93.5	94.8	98.6	105.7	102.2
Y24	94.8	105.7	100.3	95.5	97.8	96.7
Y25	87.2	109.4	98.3	104.9	110.5	107.7

Table S4 The concentration of n-alkanes in YC oilfield soils by grid sampling.

Sampling sites	Concentration of n-alkanes (mg/kg)			
	C8-C19	C20-C30	C31-C40	Total n-alkanes
Y6	50.53±8.34	942.83±67.92	246.42±34.12	1239.78±202.26
Y7	44.95±7.23	432.64±34.56	224.78±32.46	702.37±100.63
Y8	24.61±3.42	87.46±12.43	125.78±13.45	237.85±20.13
Y9	22.35±3.27	118.92±23.21	84.94±4.27	226.21±35.28
Y10	42.24±5.33	648.96±46.26	387.17±35.68	1078.37±121.24
Y11	51.39±8.21	427.53±34.56	373.49±23.75	852.41±93.27
Y12	21.58±4.35	110.33±22.37	76.71±12.45	208.62±20.65
Y13	19.6±2.37	74.38±12.34	64.29±4.26	158.27±21.79
Y14	23.03±4.26	89.32±12.93	54.09±4.30	166.44±43.24
Y15	83.43±9.24	679.76±36.24	508.55±56.90	1271.74±201.20
Y16	63.72±8.74	563.28±23.98	330.46±23.47	957.46±32.47
Y17	25.9±4.27	132.44±32.09	92.15±21.23	250.49±23.76
Y18	19.62±5.83	100.30±12.33	69.74±9.20	189.66±26.79
Y19	16.35±2.42	83.58 ±12.43	58.12±3.42	158.05±22.43
Y20	107.06±12.3	946.31±54.76	891.17±33.53	1944.54±321.22
Y21	92.46±13.53	1010.74±88.97	852.35±28.90	1955.55±45.35
Y22	15.66±3.57	198.37±19.02	198.1±18.76	412.13±32.67
Y23	126.63±11.27	2075.5±22.89	1586.56±231.23	3788.69±136.79
Y24	10.45±1.20	382.21±23.48	510.75±43.57	903.41±214.67
Y25	6.84±2.52	235.67±21.47	161.52±12.45	404.03±33.26

Table S5 The concentration of PAHs in YC oilfield soils by grid sampling.

Sampling sites	Concentration of PAHs (mg/kg)					
	Two-rings	Three-rings	Four-rings	Five-rings	Six-rings	Total PAHs
Y6	180.94±35.50	392.90±4.96	235.2±17.07	524.48±91.42	183.18±31.82	1516.70±91.41
Y7	10.48±2.06	41.42±5.52	27.36±2.96	35.59±9.24	22.16±3.96	137.19±15.85
Y8	18.02±9.20	49.30±5.09	35.52±3.57	43.84±11.87	30.40±2.60	178.08±28.55
Y9	17.21±8.98	46.33±2.86	33.07±6.68	47.96±5.46	37.78±23.97	176.35±19.81
Y10	131.21±22.63	432.96±19.49	375.88±56.56	475.19±22.19	283.55±41.65	698.79±177.15
Y11	23.23±4.28	54.35±3.68	40.36±6.22	48.67±5.52	35.78±6.54	201.39±23.46
Y12	24.92±4.77	35.20±5.53	29.39±14.59	36.21±8.26	37.90±18.37	169.62±20.76
Y13	20.70±1.06	51.43±10.35	37.55±23.89	45.35±16.94	32.77±16.63	186.8±59.75
Y14	16.12±8.08	46.54±6.48	34.08±4.45	41.14±8.32	28.86±8.32	169.74±28.95
Y15	88.89±14.66	509.77±56.86	388.07±13.80	517.87±27.44	186.11±31.98	1690.71±200.11
Y16	23.34±4.54	54.31±5.47	40.92±4.25	48.00±8.15	35.99±4.78	202.56±21.12
Y17	20.03±6.52	51.43±14.12	37.51±19.93	45.74±3.29	32.72±2.29	185.43±27.86
Y18	7.03±6.84	38.91±6.62	29.91±2.40	26.73±9.70	15.24±3.77	102.82±16.29
Y19	31.22±0.37	62.42±2.43	48.98±7.21	56.66±9.70	43.91±14.66	240.19±10.94
Y20	118.56±13.48	395.99±15.31	265.88±21.26	391.91±27.34	168.38±20.47	1340.72±137.29
Y21	101.11±3.62	334.07±29.89	218.51±29.86	260.74±7.10	141.72±19.79	1056.15±115.19
Y22	18.25±6.64	49.20±3.92	35.36±9.20	43.60±2.94	30.85±7.07	175.26±33.48
Y23	140.54±20.43	584.68±7.55	434.80±73.91	698.70±45.98	229.94±33.89	2088.66±220.37
Y24	131.14±22.12	440.76±12.70	273.45±45.83	509.02±51.82	181.36±9.84	1535.73±154.29
Y25	17.11±5.76	48.85±7.23	34.28±11.73	42.33±6.70	29.92±5.76	171.49±29.27

Table S6 Descriptive statistic TEQBap of PAHs in abandoned oil well area

PAHs	Dutch Soil Standard (μg/kg)	TEF	TEQBap of PAHs (μg/kg): abandoned time:0-5 years		TEQBap of PAHs (μg/kg): abandoned time:5-10 years		TEQBap of PAHs (μg/kg): abandoned time:10-15 years	
			Range	Mean	Range	Mean	Range	Mean
Nap	15	0.001	0.52-0.54	0.53	0.35-0.38	0.36	0.24-0.28	0.26
Acy	-	0.001	0.86-0.92	0.89	0.74-0.83	0.78	0.64-0.68	0.66
Ace	-	0.001	1.72-1.85	1.78	0.63-0.95	0.82	0.46-0.52	0.49
Flu	-	0.001	3.85-3.92	3.88	2.32-2.88	2.55	1.35-1.56	1.46
Phe	50	0.001	3.48-3.52	3.50	1.82-2.52	2.16	0.89-1.02	0.96
Ant	50	0.01	5.66-6.02	5.84	2.66-3.45	3.04	1.67-1.82	1.75
Fluo	15	0.001	1.23-1.25	1.24	0.09-1.01	1.00	0.05-0.07	0.06
Pyr	-	0.001	0.72-0.75	0.73	0.38-0.45	0.42	0.28-0.31	0.29
BaA*	20	0.1	75.64-78.99	77.34	58.23-62.58	60.41	48.27-50.32	49.80
Chr*	20	0.001	3.37-3.42	3.39	2.05-2.45	2.26	1.65-1.72	1.69
BbF*	-	0.1	271.85-283.33	277.82	145.62-183.88	162.37	126.04-128.64	127.78
BkF*	25	0.1	30.52-33.27	32.45	11.23-17.54	13.45	3.45-3.76	3.58
BaP*	25	1	426.52-466.39	446.86	224.37-266.52	246.43	120.44-122.45	121.85
InP*	25	0.1	513.65-538.73	525.34	225.84-253.20	237.46	111.25-112.23	111.75
DBA*	-	1	521.53-542.76	531.76	427.28-438.35	432.23	315.42-322.64	318.48
BghiP	20	0.01	1.01-1.03	1.02	0.06-0.08	0.07	0.02-0.04	0.03
∑PAH16	32.8	-	1410.45-1434.21	1422.27	864.54-916.89	895.72	729.88-755.78	738.36
∑PAH7	32.02	-	1385.53-1415.44	1400.48	826.73-885.43	856.08	718.65-732.47	723.91

*****indicate carcinogenic PAHs; *TEQBap*: toxic equivalent quantity based on BaP; ∑*PAH16*: sum of 16 converted PAH concentrations based on toxic equivalents of BaP. ∑*PAH7*: sum of seven carcinogenic PAHs concentrations based on toxic equivalents of BaP; *TEF*: toxic equivalency factor; *SD*: standard deviation

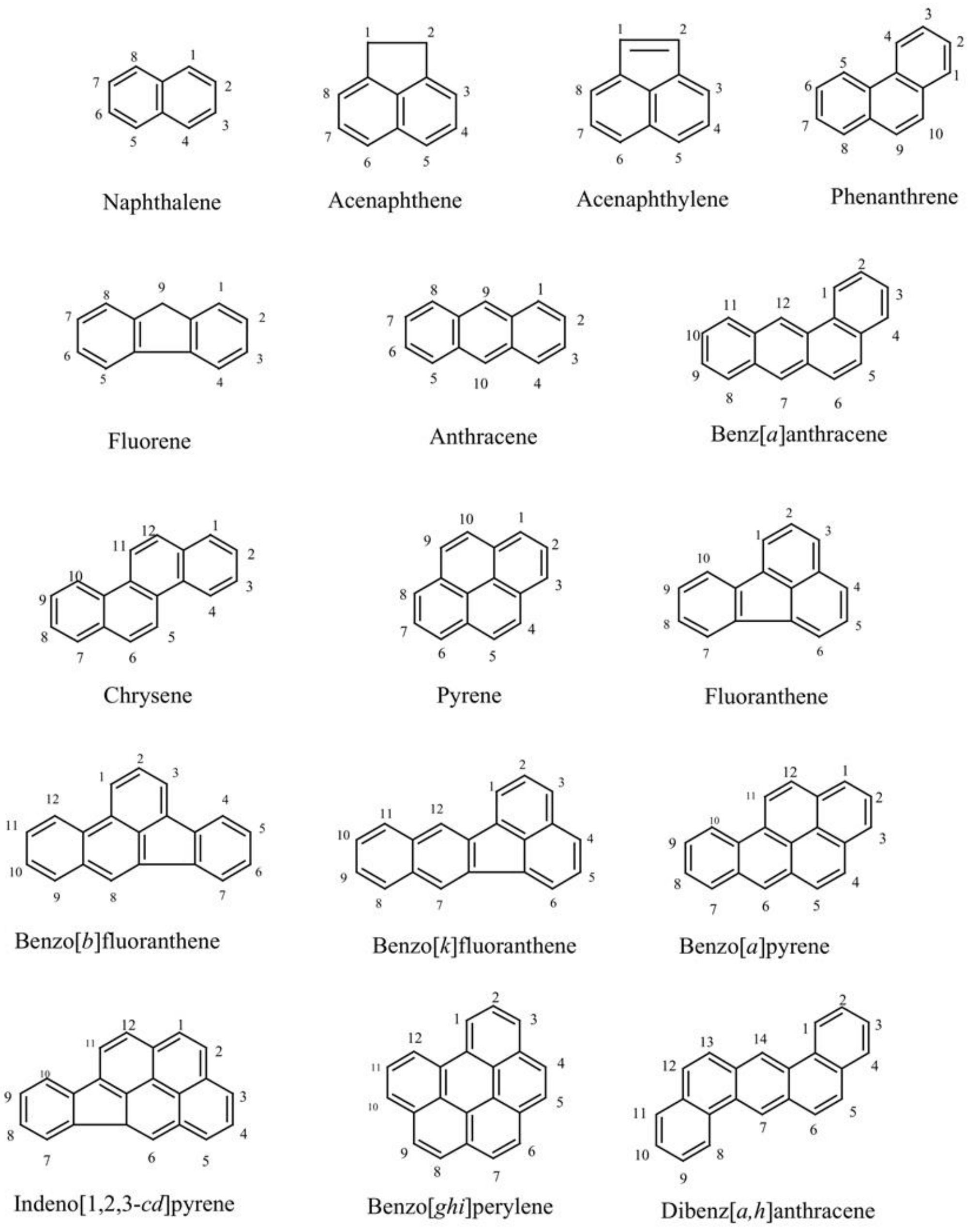

Figure S1. Structures and nomenclatures of the 16 PAHs on the EPA priority pollutant list.
